# ReadqPCR and NormqPCR: R packages for the reading, quality checking and normalisation of RT-qPCR quantification cycle (Cq) data

**DOI:** 10.1186/1471-2164-13-296

**Published:** 2012-07-02

**Authors:** James R Perkins, John M Dawes, Steve B McMahon, David LH Bennett, Christine Orengo, Matthias Kohl

**Affiliations:** 1 Institute of Structural and Molecular Biology, University College of London, Gower Street, London WC1E 6BT, UK; 2 Wolfson Centre for Age-Related Diseases, King’s College London, London SE1 1UL, UK; 3 Department of Mechanical and Process Engineering, Furtwangen University, Jakob-Kienzle-Str. 17, 78054 Villingen-Schwenningen, Germany

## Abstract

**Background:**

Measuring gene transcription using real-time reverse transcription polymerase chain reaction (RT-qPCR) technology is a mainstay of molecular biology. Technologies now exist to measure the abundance of many transcripts in parallel. The selection of the optimal reference gene for the normalisation of this data is a recurring problem, and several algorithms have been developed in order to solve it. So far nothing in R exists to unite these methods, together with other functions to read in and normalise the data using the chosen reference gene(s).

**Results:**

We have developed two R/Bioconductor packages, ReadqPCR and NormqPCR, intended for a user with some experience with high-throughput data analysis using R, who wishes to use R to analyse RT-qPCR data. We illustrate their potential use in a workflow analysing a generic RT-qPCR experiment, and apply this to a real dataset. Packages are available from http://www.bioconductor.org/packages/release/bioc/html/ReadqPCR.htmland http://www.bioconductor.org/packages/release/bioc/html/NormqPCR.html

**Conclusions:**

These packages increase the repetoire of RT-qPCR analysis tools available to the R user and allow them to (amongst other things) read their data into R, hold it in an ExpressionSet compatible R object, choose appropriate reference genes, normalise the data and look for differential expression between samples.

## Background

Several methods now exist to measure quantitatively the expression of genes within a biological sample, allowing us to compare expression between cells from different tissues, and between cells from the same tissue under different conditions. More recent technologies for this purpose include microarrays and RNA-seq. However one of the most popular remains RT-qPCR, due to its accessibility, relatively cheap price, small requisite amount of starting material and high precision [1]. Although it has a lower throughput than some other methods, technological advances in recent years have led to improvements. Through microfluidics and other technologies it is now possible to run hundreds, even thousands of RT-qPCR reactions in parallel with the same starting sample [2,3], with a high enough precision that it is frequently used in order to validate findings made through higher throughput technologies [4] (details of available technologies are provided in [5]). Its usage remains ubiquitous.

Such RT-qPCR technologies quantify gene expression by attempting to amplify a target DNA sequence, representing a gene or other biological molecule, in a query sample (the target is DNA because the RNA in the original tissue is reverse transcribed to make cDNA). The sample is placed in a well with a primer specific for the DNA sequence to be measured, necessary for amplification to begin [2]. In the case of the high-throughput RT-qPCR technologies, the sample is delivered to a number of wells in parallel, each containing a separate primer. Then a number of amplification cycles are performed for each well. A predefined threshold is set within the exponential amplification phase, when doubling of the product can be detected above background fluorescence, and the number of cycles it takes to get to this threshold is used to estimate the amount of cDNA sequence present, and thus the amount of RNA that was present in the initial tissue [2].

These values are known as quantification cycle (Cq) values (also known as threshold-cycle (Ct) values, but herein referred to as Cq, in line with the standardised nomenclature suggested in [6]). By comparing the Cq values between two samples (for example treated and untreated tissue), one can compare the amount of DNA sequence in one sample relative to the other. It is strongly recommended to normalise these raw values to account for systematic variation between samples, related to differing starting amounts of material, tissue-specific differences in transcription efficiency, and a number of other factors. This is typically achieved through the use of reference genes (endogenous control or housekeeping genes, also referred to simply as housekeepers). These are stably expressed genes that should not change in expression in response to a change in the cell’s environment, or between different cell types [1].

Assuming the reference gene exhibits stable expression across different samples, and assuming it does not show a change in expression between sample-types (i.e. between cells under different conditions/ between different cell types), the subtraction of the Cq value of the reference gene from the target gene should account for the systematic variation between samples, and allow for the expression of genes in different samples to be compared to each other directly. Furthermore it is generally recommended to combine multiple reference genes in order to reduce error, assuming their combination also shows stable expression [7].

However, it is often the case that reference genes do change in expression between sample-types, or show high stochastic variation under certain conditions [8-11]. The choice of a reference gene that shows variation between sample-types will clearly bias estimation of the expression of other genes within the samples, since subtraction of said reference gene’s expression value from a gene will lead to over or underestimation of the true expression of that gene. Similarly, a reference gene that shows a high intrinsic variation in expression under the conditions of the experiment, will lead to inflated stochastic error when estimating the true abundance of the other genes within a sample [8,12].

Several statistical methods have now been proposed to deal with the problem of reference gene selection. These methods will either select the optimal reference gene for an experiment, or a number of reference genes, whose expression values should be combined in order to generate a normalisation factor (NF), which can be used as the calibrator. The work of Vandesompele *et al.*[7] starts with a number of potential reference genes and attempts to find the best set of reference genes from this initial list (with a minimum of two, since the two most stable genes cannot be ranked). It does this by looking for the most stably expressed reference genes across all samples within an experiment, without taking into account the labelling of different sample-types. Andersen *et al.*[13] proposed a model-based approach that takes into account the overall variability of a reference gene within an experiment, and also between different sample-types. More details on these methods (amongst others) can be found in a recent paper by Chervoneva *et al.*[14], which also introduces a new method for reference gene selection, accounting for correlation between different reference genes. A summary of available software is provided in a chapter of a recently published, comprehensive book on RT-qPCR [12].

The raw-Cq value of a target gene minus that of the best reference gene is known as the *ΔCq*value. To calculate relative fold change between different conditions, the *ΔCq* value of a gene of interest in one sample type can be subtracted from its value in another sample type, in order to calculate the *ΔΔCq* value, and thus 2^−*ΔΔCq*^[15,16].

Another way the reference genes can be used to normalise the Cq results is through the adaptation of the method of Pfaffl *et al.*[17], where the efficiency of the reference gene is estimated and taken into account when normalising the other genes of interest [18].

Recently, other normalisation methods have been proposed that adapt methods originally developed for microarrays and other high-throughput genomic technologies [19-21].

Here we present two packages, ReadqPCR and NormqPCR, written in the freely available statistical computing software R (http://www.r-project.org/), [22] and available as part of the Bioconductor project (http://www.bioconductor.org/), [23]. They allow the user to read RT-qPCR data into R, deal with undetermined Cq values, find a suitable reference gene or genes for a given experiment using a method for optimal reference gene selection and normalise the data via the *ΔCq* and 2^−*ΔΔCq*^normalisation methods. The user can also use a number of existing bioconductor packages and functions to perform quality control on their data, and can check the adequacy of reference genes visually. We demonstrate the basic functionality of the packages here and provide an example work-flow, involving the different packages alongside several other well known and highly-used CRAN and Bioconductor packages, applied to a generic RT-qPCR experiment. We then present a experiment where ReadqPCR and NormqPCR have been used to analyse a RT-qPCR dataset, and take the user through the different steps that were undertaken in the analysis of the data.

## Implementation

### Typical work-flow

**Figure 1:**
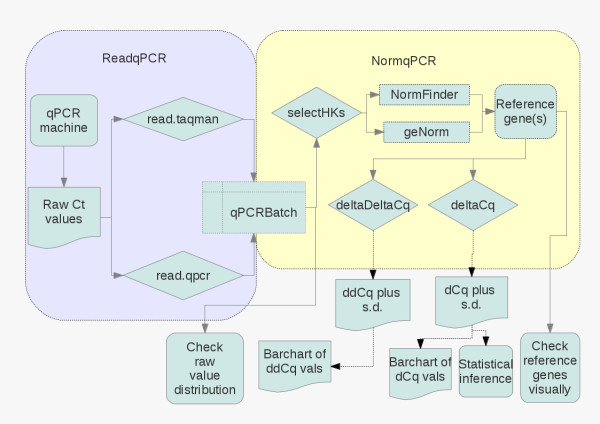
**qPCRflowChart.png.** A workflow for a typical RT-qPCR experiment making use of the two packages, showing the different steps used in the analysis, what packages are used for each step and the relevant inputs and outputs into the packages, and the names of the functions to be called. More comprehensive information, and more details about the different analyses available can be found in the package vignettes.

We have created two R packages to be used together in order to analyse RT-qPCR data. To explain the different packages to the user, we have created a work-flow, shown in Figure 1. This shows what packages should be used when, and in what order, in order to undertake a typical analysis using RT-qPCR, comparing gene expression between two conditions. For much greater detail please visit the package homepages http://www.bioconductor.org/packages/release/bioc/html/ReadqPCR.html and http://www.bioconductor.org/packages/release/bioc/html/NormqPCR.html and consult the package vignettes, which are 20-30 page synopses of the packages. Table 1 contains details of current R packages available for the analysis of qPCR data.

**Table 1:** Other R packages for RT-qPCR analyis


**Other R packages for RT-qPCR analyis**					
**Package**	**Availability**	**Data**	**Quality**	**Normalisation**	**Reference Gene**
**Name**		**Import**	**Control**		**Selection**
HTqPCR	http://www.bioconductor.org/packages/release/bioc/html/HTqPCR.html	Y	Y	Y	N
qpcR	http://cran.r-project.org/web/packages/qpcR/index.html	N	N	Y	N
qpcrNorm	http://www.bioconductor.org/packages/release/bioc/html/qpcrNorm.html	N	N	Y	N
SLqPCR	http://www.bioconductor.org/packages/release/bioc/html/SLqPCR.html	N	N	Y	Y
ddCt	http://www.bioconductor.org/packages/release/bioc/html/ddCt.html	Y	N	Y	N

### RT-qPCR packages

#### Data capture

Firstly the user will run the experiment. This will produce output, including amongst other things, the names of the genes being measured, and the Cq values for each gene in each sample. It is important to adjust the baseline correctly using the appropriate software if necessary. Depending on the technology used, there may or may not be a function in the ReadqPCR package that can read the raw (text) output from the machine, and upload it directly into R. If such a function is not available, the output must be converted into a simple tab-delimited format, using spreadsheet software or a scripting language (more details in the package vignette), which can then be uploaded into R via ReadqPCR. This will use the names of the target genes (or other biological entity to be measured, such as miRNA), the sample names and the raw Cq values to generate an R-object of class “qPCRBatch”, an extension of the “expressionSet” class, which is intended to be the standard container for high-throughput assays and experimental meta-data in Bioconductor [24]. If the input file contains data on the positions of the wells in which the experiments were performed, this will also populate the “qPCRBatch” object. A “qPCRBatch” can contain an indefinite number of different conditions, from one to as many as the R instance can handle. More than one input file can be uploaded into a single “qPCRBatch”, as long as all the input files contain either the same sample names, or same feature identifiers (such as gene names).

**Figure 2:**
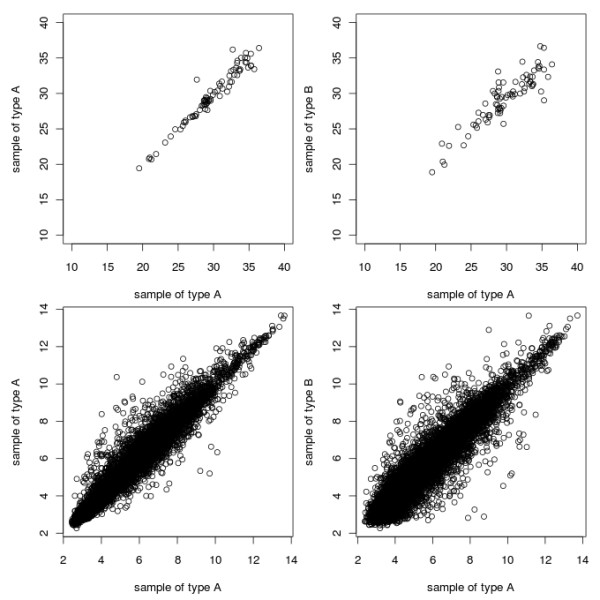
**qPCRpairsPlots.png.** Plots of Cq values. The top row plots show the difference between the expression values for genes measured by RT-qPCR technology, between samples of the same sample-type (top left), and between different samples (top right). The bottom row plots show the same, but for genes measured using microarrays The difference in numbering on the different axes is due to the different type of data returned by the different technologies. Datasets used are from the ALL package (microarray data) and the example dataset available in the ReadqPCR package (RT-qPCR data).

Once the raw data has been loaded into R and a “qPCRBatch” object has been generated, the distribution of Cq values for each sample can be compared in a pair-wise manner, using the pairs() function or the mva.pairs() function from the affy package [25] as a quality control step to identify outlying samples. This will not always be sensible; for an experiment investigating a small number of genes, with the majority of them changing between conditions, the pair-plots are likely to show little correlation between different sample types. This is unlike microarrays, where often the majority of genes being estimated do not change between sample-type. This contrast between RT-qPCR and microarray pairs-plots is shown in Figure 2. However pair-plots can still be useful for comparing different samples within the same sample-type, i.e. biological replicates, and for a visual way to compare within-sample variation with the variation resulting from the different conditions being compared (also shown in Figure 2, top row).

Though not shown in the work-flow, ReadqPCR also allows the user to deal with technical replicates by (optionally) calculating the arithmetic mean or median of the raw Cq values for the technical replicates of a given gene.

Missing values can be handled by our packages in a number of ways, as detailed in the vignette. We advise the user to take care when using these functions as missing value imputation may lead to inflated stability values for reference genes.

#### Optimal reference gene selection and normalisation

The next step is to find the best reference genes for a given experiment. NormqPCR currently implements two methods for this puprose: a pair-wise stability based method (geNorm) [7], which compares the expression of possible pairs of different reference genes, eliminating “bad” genes in a step-wise manner, and a model based method (NormFinder) [13], which takes into account variability of the reference genes between sample types, as well as overall variability in expression. In the case of the former, a minimum of two reference genes can be found, in the case of the latter, a minimum of one. If the user already knows what reference gene(s) to use, this step can be skipped. These methods are implemented in NormqPCR using the selectHKs() function, which can take either “geNorm” or “NormFinder” as an argument. It is important to note that although the selectHKs() function can accept 2^*Cq*^ or Cq values, it is important to specify this with the “log” argument when calling the function.

Once the user has identified what reference gene or genes to use, using one of the above methods, the next step is to subtract the Cq values for the reference gene (or in the case of more than one, the mean of the Cq values) from the other genes in each sample, in order to normalise them (produce the *ΔCq*value) and allow a direct comparison of gene expression between different sample-types (by calculating the 2^−*ΔΔCq*^value). In the case of more than one reference gene being selected, a normalisation constant (NC) will be calculated, as the arithmetic mean or median of the Cq values of the reference genes. Under the assumption that the RT-qPCR efficiency is equal to 2, this is equivalent to using a normalisation factor, as we show below.

In [18] they propose using a normalisation factor calculated as the geometric mean of the relative quantity (RQ) values of the reference genes, based on previous work [7,17]. RQs for some gene *j* are computed as

(1) $$RQ_{j}=E_{j}^{Cq_{j}}$$

Where *E*_*j*_is the RT-qPCR efficiency for gene *j*, and the normalisation factor is calculated as

(2) $$\mathrm{NF}=\sqrt[{f}]{\overset{f}{\underset{p=1}{\prod }}RQ_{p}}$$

where *p*=1,…,*f*indicates the *f * reference genes we have chosen. Thus following the method described in [18], the Cq values of target genes can also be converted to RQ, and divided by the NF to make normalised relative quantitiy (NRQ) values. So for a given gene *j* one obtains

(3) $$\mathrm{NR}Q_{j}=\frac{RQ_{j}}{\mathrm{NF}}$$

However, assuming *E*=2 for all target genes and reference genes, we can also use the arithmetic mean of the reference gene Cq. This can be seen by rewriting equation (3) as

(4) $$\underset{2}{\log}\left(\mathrm{NR}Q_{j}\right)=\underset{2}{\log}\left(2^{Cq_{j}}\right)-\underset{2}{\log}\left(\sqrt[{f}]{\overset{f}{\underset{p=1}{\prod }}2^{Cq_{p}}}\right)$$

leading to

(5) $$\underset{2}{\log}\left(\mathrm{NR}Q_{j}\right)=Cq_{j}-\frac{1}{f}\overset{f}{\underset{p=1}{\sum }}Cq_{p}$$

This is what we refer to as the *ΔCq*_*j*_value and so

(6) $$\mathrm{NR}Q_{j}=2^{\left[Cq_{j}-\frac{1}{f}\overset{f}{\underset{p=1}{\sum }}Cq_{p}\right]}$$

**Figure 3:**
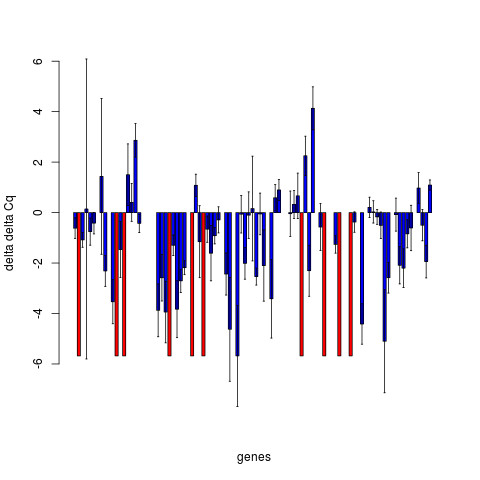
**qPCRdDCq.png.** Relative (log2) fold change between case and control for a number of genes. Blue bars show mean, error bars ± 1 standard deviation Red bars show instances where the presence of the transcript has been detected in the one condition, but not in the other, and as such no fold change or standard deviation (of fold change) can be calculated.

To perform relative quantification between different sample types, the 2^−*ΔΔCq*^ values should be calculated by subtracting the *ΔCq*(i.e. the log_2_(NRQ) from equation 5) value for a given gene for the case sample from the control sample, i.e. $2^{\left[\mathrm{\Delta C}q_{\mathrm{control}}-\mathrm{\Delta C}q_{\mathrm{case}}\right]}$, also written as $2^{-\left[\mathrm{\Delta C}q_{\mathrm{case}}-\mathrm{\Delta C}q_{\mathrm{control}}\right]}$. 2^−*ΔΔCq*^ values can be calculated using the deltaDeltaCq() function in NormqPCR. This will return a list of all target genes with their corresponding values. These results can also be plotted as bar charts in order to show more clearly what genes are showing differential expression, and the range of error. An example, taken from the real data analysis presented below shows the *ΔΔCq* values, although the 2^−*ΔΔCq*^values might also have been plotted (Figure 3). Standard deviations are calculated following the protocol presented in [15], using the “same well” method, presented in table 2 of this paper: the standard deviation of the differences between Cq values of the target gene and the housekeeper, for the different samples, is calculated. The single well method from table 1 of the same paper is also available by calling deltaDeltaCt() function with the argument “paired = TRUE”. In both cases, the subtraction of the calibrator is treated as the subtraction of an arbitrary constant, and so does not increase the estimated error [15].

One inherent problem with RT-qPCR is that some values are undetermined. This occurs when the amplification of certain products is not detected above the level of noise, typically within 40 cycles, and is interpreted as absence of target transcript. In the case that values are undetermined in one set of samples and not the other (i.e. in case but not control, or vice-versa), the deltaDeltaCq() function outputs a “+” or “-” for the fold change, though if a user wishes to impute their own value to replace the “+”/“-” they can do so easily (though we advise caution when doing so).

The user may wish to perform statistical tests for differential expression, perhaps using the limma package [26], the base R function wilcox.test() or the rowttests() functions in the genefilter package. It is recommended to use the deltaCq() function and use the resulting “qPCRBatch” object for this analysis. This object will contain the *Δ*Cq values for each gene in each condition, which we expect to be normally distributed.

**Figure 4:**
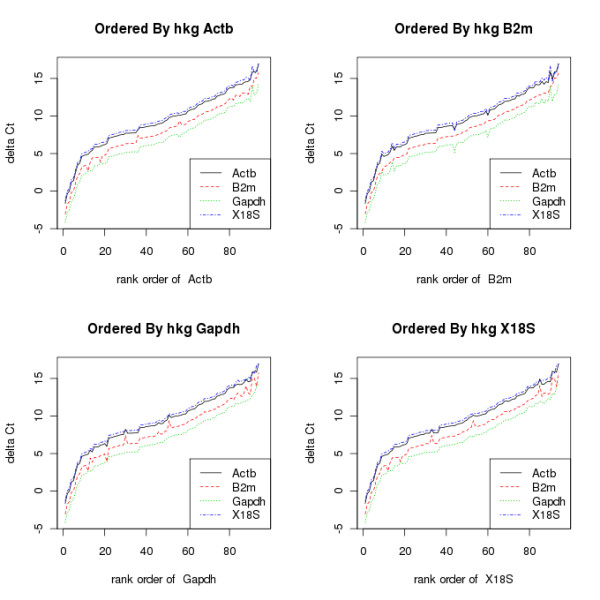
**qPCRplotByRef.png.** These show the mean *ΔCq*values for all values following normalisation by a reference gene, in each case ordered by differing reference genes. In this case B2m appears to show the least correlation with the others, suggesting it might be removed when calculating the normalisation factor.

The *ΔΔCq*step can also be exploited as another method to visualise the stability of the reference genes. By calculating *ΔΔCq* for each reference gene, and plotting these, in ascending order as normalised by a nominal reference gene, the user can see whether some reference genes show more similar expression to each other, and whether other genes stand out. This is shown in Figure 4, which contains graphs of the *ΔΔCq* values for all the genes in an experiment, each ordered by a different reference gene. In each graph, the values are ordered from lowest to highest.

## Results and discussion

ReadqPCR and NormqPCR were used to analyse a real dataset, investigating the effects of UVB radiation on gene expression in skin by comparing UVB radiated skin to untreated. Full experimental details are available in [27], including descriptions of the samples used, their processing, storage conditions, and the experimental set-up. Additional file 1 shows the series of commands that were used to analyse the data set. In brief, 8 biological replicates were produced for each sample-type, and each sample was analysed using Taqman array cards, 384 well microfluidic arrays produced by Applied Biosystems. The expression of 96 genes was measured for each sample, 92 target genes and 4 reference genes: Beta-actin, beta-2 microglobulin, GAPDH, and 18S ribosomal RNA. Each card contained four samples, two case (UVB treated skin sample) and two control (untreated skin sample).

The SDS output file, as obtained from the PCR system, was loaded into R using the ReadqPCR package, populating a “qPCRBatch” object (S1-block A). NormqPCR was then used to find the optimal reference genes, using geNorm (S1-block B). This indicated that four reference genes were required, since inclusion of a fourth gene leads to a reduction in variation. NormFinder could also have been used to select the reference genes.

The arithmetic mean of the *Cq* values of the four reference genes was then calculated to produce a normalisation constant, and this was subtracted from each of the Cq values of the target genes to calculate the *ΔCq* values (S1-block C). Then Mann-Whitney U-tests were used on the *ΔCq*values in order to calculate a p-value for differential expression between case and control (S1-block D). Forty two genes were shown to be differentially expressed (p-value <0.05). Multiple testing correction was also performed, using the method of Benjamini Hochberg [27], (S1-block E), leaving 39 genes significant with an estimated false discovery rate (FDR) of 0.05.

Finally, 2^−*ΔΔCq*^ were produced, for all genes (S1-block F) and the corresponding *ΔΔCq*values were plotted with the corresponding error bars representing +/- one standard deviation (S1-block G). In the case that Cq values were NA in case, but where values were obtained for control, and vice versa (i.e. NAs for control but values for case), the bars were given a different colour and a height of the maximum fold change in the experiment, and no error bars were plotted (Figure 3 (qPCRdDCq.png)).

## Conclusions

ReadqPCR and NormqPCR provide tools for uploading RT-qPCR data into R, look for the optimal reference genes, and normalise the data using the *ΔΔCq*method. It has already been used by an experimental group to calculate differential expression using Taqman RT-qPCR data [27].

These packages, implementing popular optimal reference gene finding algorithms in the widely-used statistical software for genomic analysis, R, represent an important contribution to the RT-qPCR community, and increase the available options for the analysis of this type of data.

## Availability and requirements

### Project name

ReadqPCR/NormqPCR - R packages for the reading, quality checking and normalisation of RT-qPCR quantification cycle (Cq) data.

### Project home page

http://www.bioconductor.org/packages/devel/bioc/html/ReadqPCR.html

http://www.bioconductor.org/packages/devel/bioc/html/NormqPCR.html

### Operating system(s)

Platform independent

### Programming language

R (http://www.r-project.org)

### Other requirements

Bioconductor

### License

LGPL-3

## Competing interests

The authors declare that they have no competing interests.

## Authors’ contributions

JP: Wrote the manuscript. MK & JP: Conceived the project and developed the software packages. JMD, SBM and DLHB helped in testing the package, producing data for testing and providing critical feedback. CO: Contributed to writing and revising the manuscript, and helped plan the work. All authors read and approved the final manuscript.

## Supplementary Material

Additional file 1: qPCRPaperScript.R. and UVB.txt. Click here for file
